# Reduction in ventialator associated pnermonia rates in an Indian ICU as an outcome of prevention bundle implementation

**DOI:** 10.1186/1753-6561-5-S6-P69

**Published:** 2011-06-29

**Authors:** N Jaggi, P Sissodia, E Narayan

**Affiliations:** 1 Labs & Infection Control, Artemis Health Institute, Gurgaon, Haryana, India

## Introduction / objectives

The impact of bundled infection control practices introduced in the Intensive care units (ICU) is analyzed to identify the most effective infection control practice in reducing Ventilator Associated Pneumonia (VAP) rates.

## Methods

VAP data over a period of 24 months (Jan’09 to Dec’10) in a Tertiary care Indian ICU was retrospectively analyzed. The impact of each intervention of prevention bundle introduced in Dec’09 was critically evaluated on VAP rates in the subsequent year 2010.

## Results

The VAP rates over the entire study period varied between 0 and 30.9/1000 ventilator days. The VAP rate for the year 2009 was 9.72 (22 VAP infections in 2263 ventilator days) which reduced to 3.43 (11 VAP infections in 3202 ventilator days) in the following year 2010 as a result of the interventions. The study showed hand hygiene compliance of the healthcare workers and length of stay of patient as the major risk factors associated with VAP. The most effective intervention strategies analyzed were head of bed elevation (p < 0.0001), sub glottic suction (p < 0.0001), hand hygiene compliance of healthcare workers (p < 0.0001) and daily assessment of weaning and extubation for ventilated patients (p < 0.0001). The other measures like closed endotracheal suctioning (p= 0.257), humidification with heat and moisture exchangers (p=0.091), chlorhexidine mouthcare every 2 hours (p=0.002) and routine drainage of ventilator circuit condensate (p=0.0112) proved to be less significant.

## Conclusion

Head of bed elevation, hand hygiene compliance of healthcare workers, sub glottic suction and daily assessment of weaning and extubation for ventilated patients contributed as the most effective infection control practices in VAP prevention.

## Disclosure of interest

None declared.

